# Quality of life and psychological problems in patients undergoing neurological rehabilitation

**DOI:** 10.4103/0972-2327.44557

**Published:** 2008

**Authors:** Anupam Gupta, S. Deepika, A. B. Taly, Abhishek Srivastava, Vishal Surender, Murali Thyloth

**Affiliations:** Department of Psychiatric and Neurological Rehabilitation, National Institute of Mental Health and Neuro Sciences (NIMHANS), Bangalore, India; 1 Department of Neurology, National Institute of Mental Health and Neuro Sciences (NIMHANS), Bangalore, India

**Keywords:** Neurological dysfunction, psychological adjustment difficulty, quality of life

## Abstract

**Aim:**

To assess the quality of life (QoL) and prevalence of psychological problems (PP) in patients with neurological illness, and their correlation with functional abilities.

**Materials and Methods:**

Prospective cross-sectional study conducted in the neurological rehabilitation unit of tertiary research hospital in 30 consecutive hospitalized patients (21 men), age 16-55 years (34.63±11.87).

**Outcome Measure:**

WHOQoL-BREF was used to assess QoL. The prevalence of PP was recorded using Hospital Anxiety Depression Scale (HADS) and General Health Questionnaire (GHQ-12). QoL and HADS scores were correlated with functional abilities using mean Barthel Index (BI) Score.

**Results:**

The duration of illness was three to 30 months (10.63±7.83) and their primary diagnoses were stroke 12, traumatic spinal cord injury seven and non-traumatic spinal cord lesion 11. Twenty-two patients qualified for GHQ-12 caseness, with 15 patients having distress (score ≥15) and seven having severe problem and psychological distress (score≥20). Twenty five patients had abnormal anxiety and 17 had abnormal depression on HADS (abnormal = 8-21), with moderate to severe anxiety and depression (scores≥11) in nine and three patients respectively. The mean WHOQoL-BREF transformed scores (on WHOQoL 0-100 scale) were (38.83±8.02), (50.76±9.79), (48.53±18.46) and (49.13±10.63) in physical, psychological, social, and environmental domains respectively. The social domain of QoL had significant correlation (*P*<.05) with functional abilities.

**Conclusion:**

Patients with neurological disorders requiring inpatient rehabilitation have impaired QoL that affects all domains of life. There is high prevalence of psychological problems, including anxiety and depression. The social domain of QoL adversely affected functional abilities, but the correlation between PP and functional abilities was insignificant.

## Introduction

The World Health Organization (WHO) defined QoL as an ‘individual's perceptions of his position in life, in the context of the culture and value system in which he lives and in relation to his goals, standards, and concerns.’[1] A number of recent studies have focused on long term QoL after stroke[2–5] and Spinal Cord Injury[6–9] and the correlation between the QoL and functional abilities of the patient after stroke.[10–11]

The direct effects of the disease, as well as the side effects of the treatment, may influence QoL in patients with neurological illnesses like stroke or spinal cord injury (SCI). The major medical consequences of stroke concern different physical and psychological aspects such as loss of sense, palsy, and disturbance of body image, depression and change in patient's role, all of which affect health related QoL.[12]

Low scores in all QoL domains, namely physical, psychological, social and environmental, have been observed up to three years after a stroke, with significant correlation with the functional abilities of the patients. It has also been observed that depression has a worse effect on QoL and the functional abilities of these patients.[4,5] Psychological problems like depression and anxiety in patients with neurological illness require monitoring alongside QoL assessments, especially across longitudinal studies, in order to examine the relationship between QoL perceptions and emotional state.[13]

Difficulties in adjusting to chronic illness may lead to decreased QoL, poor self care and multiple medical problems.[14] Even more distressing is the estimate that persons with spinal cord injury commit suicide two to six times more frequently than the general population.[15–17] The frequency of major depression after acute spinal cord injury, regardless of level, reaches about 20% measured by objective psychological testing.[18–21] Thus, early diagnosis and treatment of clinically depressed patients must be inherent to any rehabilitation, as acutely injured patients with major depression do poorly in rehabilitation.[12,22] QoL is also adversely affected in patients following spinal cord injuries (SCI). Patients who have been living with SCI report a better QoL, as compared to those recently injured, suggesting an adaptive process operating over a long period.[8] The presence of complicating medical problems, such as severe pain, spasticity, and incontinence, seem to have more negative effects on QoL than the extent of SCI.

The present study was conducted to assess the QoL and prevalence of psychological problems in patients with neurological illness, admitted for rehabilitation, and to observe the correlation between QoL, anxiety and depression with functional abilities/disability of these patients.

## Materials and Methods

Thirty patients (M:F :: 21: 9), in the 16-55 age group (34.63±11.87), admitted with stroke or spinal cord injury to the neurological rehabilitation unit from December 2006 to April 2007 consecutively, were included in the study, after taking written informed consent. The duration of illness in the study (n=30) varied between three and 30 months (10.63±7.83). Twelve patients with stroke had 3-30 months' duration of illness (13.17±9.94) and 18 myelopathy patients had 4-21 months' duration of illness (8.94±5.76). Stroke patients with global or sensory aphasia and with cognitive deficits were excluded from the study.

All the patients were assessed using self-reported and observer rated questionnaires and interviews. These included HADS[13] [Hospital Anxiety and Depression Scale], GHQ-12[23] [General Health Questionnaire-12] and WHOQoL-BREF[24] (World Health Organization-Quality of Life-Geneva 1996). Functional ability of the patients was assessed using Barthel Index Score.[25] WHOQoL transformed mean scores in all four domains, namely physical, psychological, social and environmental, were correlated with mean Barthel Index score at the time of admission of the patients, in the neuro-rehabilitation unit and the results were analyzed.

### Hospital Anxiety and Depression Scale (HADS)

The HADS was originally developed for use in hospital settings. It was designed as a self-completed questionnaire to assess a patient's anxiety and depression, whilst in in-patient care, according to two sub-scales. The Anxiety and Depression scales both comprise seven questions rated from a score of 0 to 3, depending on the severity of the problem described in each question. The two sub-scales can also be aggregated to provide an overall anxiety and depression score. The anxiety and depression scores are categorized as given below:

Aggregate Score

Interpretation

0-7→ Normal

8-10→ Mild anxiety/depression

11-14→ Moderate anxiety/depression

15-21→ Severe anxiety/depression

### General Health Questionnaire 12 (GHQ-12)

The General Health Questionnaire (GHQ) is a self-administered screening test, designed to identify short-term changes in mental health (depression, anxiety, social dysfunction and somatic symptoms). It is a pure state measure, responding to how much a subject feels that the present state ‘over the past few weeks’ is unlike his/her usual state. It does not make clinical diagnoses and should not be used to measure long-standing attributes. The GHQ focuses on the person's ability to carry out ‘normal’ functions and the appearance of any new disturbing phenomena.

Scoring - Likert Scale 0, 1, 2, 3 from left to right.

12 items, 0 to 3 each item

Score range 0 to 36.

Scores vary by study population.

Scores about 11-12 typical.

Score >15 - evidence of distress

Score >20 - suggests severe problems and psychological distress

### WHOQoL-BREF

The WHOQoL-100 allows a detailed assessment of each individual facet, relating to quality of life. It has four domains: physical health, psychological, social relationship and environment. It contains 26 questions. To provide a broad and comprehensive assessment, one item from each of the 24 facets (from the four domains) contained in WHOQoL-100, has been included. Out of 26 questions, seven are related to physical health, six to psychological status, three to social support and eight to the environment domain of the patient. In addition, two items from the overall QoL and general health facet have been included. After drawing raw scores from the questionnaire, they are converted into the transformed score with the help of a table, which is considered the final score in that particular domain. As no cut-points exist for good and poor QoL measured by WHOQoL-BREF, poor quality of life is indicated by numbers pointing to 0 and high quality is characterized by numbers pointing to 100.

### Barthel Index

Barthel Index is one of the most widely used outcome measure for functional abilities of patients in rehabilitation settings. It is a 10-item scale, which assesses patients’ ability for feeding, bathing, grooming, dressing (both upper and lower half), bladder and bowel care, personal toilet, transfers, stair negotiation abilities and mobility (with or without orthoses, wheel chair and assistive devices) by himself/herself. It is a 100-point scale, with the higher score suggesting more functional independence of the patient.

### Analysis

The data was analyzed using SPSS 15.0. Descriptive analysis was done using range, mean and standard deviation. Spearman correlation co-efficient was used to assess the correlation between Anxiety and Depression scores; mean Barthel Index score at admission and Pearson correlation co-efficient were used to assess the correlation between physical, psychological, social and environmental domains of QoL and mean Barthel Index scores at admission and duration of illness. Paired students’ t test was used to assess the functional recovery in all the patients, by comparing mean Barthel index scores at admission and discharge.

## Results

Thirty patients (M : F :: 21: 9), in the 16-55 age group (34.63±11.87), were included in the study. Primary diagnosis of the patients included is reported in Table 1. Duration of illness at the time of admission varied from 3-30 months, with a mean of 10.63±7.83 months. The duration of illness was correlated with all four domains of QoL in the patients. The social domain of QoL had significant correlation with the duration of illness (*P* =0.026), while the other domains namely physical, psychological and environment had insignificant correlation (*P* =0.305, 0.824 and 0.057 respectively).

**Table 1:** Clinical condition of the patients

Diagnosis	No. of patients
Left hemiplegia	11
Right hemiplegia	1
Traumatic spinal cord lesion	7
Spinal cord injury	
Pott's spine	4
Transverse myelitis	4
Spinal tumor	1
Ossified post. longitudinal ligament	2
Total	30

Out of 18 myelopathy patients, eight had total paraplegia below the level of lesion, with no sacral sparing (ASIA A); seven had sensory but no motor recovery below the level of lesion (ASIA B) and three patients had some motor power (ASIA C) at the time of admission, according to the ASIA impairment scale.[26] All stroke patients were ambulatory at the time of admission, with varying dynamic deformities on the affected side. Seven stroke patients had periarthritis shoulder, five patients had flexed wrist/clenching of hand in the upper limb and five others had dynamic equines deformity. Two patients had already received AFO's (ankle-foot orthosis) and five were using cane for walking at the time of admission.

All myelopathy patients had bladder involvement and underwent urodynamic study (UDS), as per the protocol routinely followed in the unit. Thirteen myelopathy patients had overactive detrusor, with six patients without sphincter dyssynergy and seven patients with dyssynergy. Five patients had under active detrusor. All stroke patients were continent for bladder, although three patients had complaints of increased frequency and urgency of micturation. UDS was not advised to any stroke patient.

One patient with right hemiparesis had Broca's aphasia and two patients with left hemiparesis had dysarthria. One patient with brainstem stroke had swallowing disorder and was on N-G tube feeding at the time of admission. They were treated by a speech pathologist, one of the members of the rehabilitation team in the unit.

General Health Questionnaire (GHQ-12) was used to assess the general health of the person in the past few weeks. Twenty two patients (73.33%) in the study qualified for caseness, according to GHQ-12, with 15 patients (50%) scoring more than 15, denoting distress, and seven patients (23.33%) scoring more than 20, showing severe problem and psychological distress over the past few weeks.

HADS was used in the case of all the patients, to assess psychological problems. This scale elicits the anxiety and depression in the preceding two weeks. Overall, 25 patients (83.33%) showed abnormal anxiety according to HADS scale; 16 (53.33%) showed mild anxiety and nine (30%) showed moderate to severe anxiety. Seventeen patients had features of depression, on the HADS scale; 14 (46.67%) showed mild depression and three (10%) showed moderate to severe depression. Spearman rank test was used to observe the correlation between anxiety and depression and Barthel Index score, a measure of functional abilities of the patients. It was found to be insignificant (*P* =.214 and .823, for anxiety and depression respectively).

Mean Barthel index scores at the time of discharge were compared with the mean admission scores, to assess the functional recovery in all the 30 patients during their stay in the rehabilitation unit. Mean admission score was 41.67±16.27 (20-80), whereas mean discharge score was 64.16±16.40 (40-90). Significant functional recovery was observed (*P* = .000) with rehabilitation intervention in the study using paired students’ t test.

The World Health Organization Quality of Life (WHOQoL-BREF) scale is the abbreviated version of WHOQoL-100, which assesses a person's quality of life across four domains, namely physical, psychological, social and environmental. Quality of Life assessment of all patients in the study was done administering the same scale. Their raw scores were calculated and converted into transformed scores. Mean scores were 38.33±8.02, 50.76±9.79, 48.53±18.46 and 49.13±10.63 in the physical, psychological, social, and environmental domains respectively.

Transformed scores in each domain were correlated with mean Barthel Index score. Patients had low scores in all the four domains, namely physical, psychological, social and environmental of quality of life, with social domain having the most significant correlation with the functional abilities of the patients (*P*<0.05) [Table 2].

**Table 2:** Correlation between mean barthel index score and domains of WHOQoL

		WHOQoL-BREF mean transformed scores (n=30)			
		
		Physical	Psychological	Social	Environmental
Mean BI score at admission	41.17	38.83	50.76	48.53	49.13
Pearson correlation Co-efficient Sig.(2-tailed)		0.233	0.745	0.017	0.077

## Discussion

Patients with chronic conditions like stroke and myelopathy usually have lower QoL, as compared to the general population. The disability and handicap caused by these lesions also causes psychological problems, which may manifest as anxiety and/or depression.

Hospital Anxiety and Depression Scale showed that 25 patients (83.33%) had abnormal anxiety and 17 patients (57%) had abnormal depression in the present study. Moderate to severe anxiety and depression were found in nine (30%) and three (10%) patients respectively. These patients were treated with psychotherapy alone/along with anxiolytic or anti-depressant medication, after evaluation by a psychologist and psychiatrist, both of whom were a part of the rehabilitation team. The General Health Questionnaire (GHQ-12) was used to assess changes in mental health (depression, anxiety, social dysfunction and somatic symptoms) in the patients. It showed that 15 (50%) patients in the present study had distress, while seven (23.33%) patients had severe distress and psychological problems.

It is important to examine the prevalence of depression after SCI, because it is often associated with the occurrence of secondary complications such as pressure sores, urinary tract infections, and contractures.[27] Dryden *et al*. (2005) observed that 28.9% of the patients with spinal cord injury required treatment for depression. According to the authors, individuals at the highest risk were those with a preinjury history of depression, a history of substance abuse or permanent neurological deficit.[28] In our study, none of the patients had history of depression prior to neurological illness, but 11 patients had history of substance abuse in the form of smoking cigarettes/bidis (seven patients of stroke and four patients with myelopathy) for more than five years, and five patients were consuming alcohol for more than five years. Five smokers and two alcohol users required medication (anti-depressant) for the management of their anxiety/depression.

The National Institute of Mental Health in USA estimated that 10 to 27 percent of stroke survivors experience major depression, and an additional 15 to 40 percent have symptoms of depression within two months following the stroke. The average duration of major depression poststroke is just under one year. In our study, one stroke patient (8.33%) had moderate to severe depression and required medication, whereas three stroke patients (25%) had moderate to severe anxiety (according to HADS) and required psychotherapy and anxiolytic medication. Thus, the prevalence of depression and anxiety in this study was in accordance with the earlier studies.

Early diagnosis and intervention (treatment of depression) is imperative, as depression adversely affects the functional abilities of the patient in the post stroke period and improvement in depressive symptoms may accelerate functional recovery, although the level of physical functioning achieved post stroke is also determined by neurological and cognitive factors.[29] In the present study, the correlation between anxiety and depression and functional abilities of the patient was not significant (*P*>0.05), contrary to the study by Lo *et al*. (2008), in which the authors found significant correlation between depression and handicap in the post-stroke period.[11]

Measuring QoL is useful in gaining a better understanding of patients’ reaction to the illness and for the development of therapeutic processes, as well as in monitoring the efficacy of medical care both in the acute phase as well as in the long term follow up. There is a need to incorporate measures such as QoL, in addition to traditional scales of impairment, to assess the impact of chronic neurological illnesses and their treatment, on the life of the patients.[30] Even those with only mild consequences of stroke have low QoL,[31] which may persist during long term follow-up of the patients.[3,5,32] Mean scores (WHOQoL-BREF) in the present study were suggestive of low QoL in all domains, namely physical, psychological, social and environment. It was a cross-sectional study and the follow-up data of the patients were not analyzed. However, long term follow-up studies with same neurological illnesses have suggested persistent low QoL.

Each QoL domain scores were correlated with the functional abilities of the patients (using Barthel Index scores), to observe how they affect the disability of the patients. Barthel Index score is one of the most commonly used scales for measuring functional outcome in the patients with neurological illness and disability in different rehabilitation settings. Wilkinson *et al*. (1997) concluded that Barthel Index can be used as the standard outcome measure for populations of stroke patients, for long term follow up.[10]

Significant relationship was found between the social domain of QoL and mean Barthel Index score (p<0.05). Social domain consists of personal support, sexual activity and personal relationship of the patient. Some studies[5,6] have observed the same results with significant correlation between the social domain of QoL and the functional abilities of the patients; others[32,33] have shown insignificant correlation between quality of life and neurological impairment and functional abilities of the patients with similar illnesses. The findings in the present study emphasize the fact that patients with stroke and myelopathy need strong family and social support to motivate and assist them in attaining the goal of functional independence, consistent with the severity and deficits as a result of neurological illness. Sexual rehabilitation is essential, along with physical and psycho-social rehabilitation, and it should be an integral part of any rehabilitation program.

## Conclusions

There is high prevalence of psychological problems including anxiety, depression and social dysfunction in patients undergoing neurological rehabilitation. Quality of life of these patients is low and it affects all domains of the life of the patients. Functional abilities are adversely affected in patients with neurological dysfunction. The correlation between social domains of quality of life and functional abilities was observed to be significant in the study.

### Limitations of the study

This study was performed on a very small sample of patients. Studies with larger sample size and follow-up will help us understand the QoL issues and the issue of prevalence of psychological problems in patients with neurological dysfunction.
